# Nanosensor-Enabled Detection and Identification of Intracellular Bacterial Infections in Macrophages

**DOI:** 10.3390/bios14080360

**Published:** 2024-07-25

**Authors:** Aritra Nath Chattopadhyay, Mingdi Jiang, Jessa Marie V. Makabenta, Jungmi Park, Yingying Geng, Vincent Rotello

**Affiliations:** Department of Chemistry, University of Massachusetts Amherst, 710 North Pleasant Street, Amherst, MA 01003, USA; aritranathch@umass.edu (A.N.C.); mingdijiang@umass.edu (M.J.); jmakabenta@umass.edu (J.M.V.M.); jungmipark@umass.edu (J.P.); ygeng@umass.edu (Y.G.)

**Keywords:** intracellular infection, macrophage infection, cell surface phenotypic changes, chemical nose sensing, synthetic polymeric sensor array

## Abstract

Opportunistic bacterial pathogens can evade the immune response by residing and reproducing within host immune cells, including macrophages. These intracellular infections provide reservoirs for pathogens that enhance the progression of infections and inhibit therapeutic strategies. Current sensing strategies for intracellular infections generally use immunosensing of specific biomarkers on the cell surface or polymerase chain reaction (PCR) of the corresponding nucleic acids, making detection difficult, time-consuming, and challenging to generalize. Intracellular infections can induce changes in macrophage glycosylation, providing a potential strategy for signature-based detection of intracellular infections. We report here the detection of bacterial infection in macrophages using a boronic acid (BA)-based pH-responsive polymer sensor array engineered to distinguish mammalian cell phenotypes by their cell surface glycosylation signatures. The sensor was able to discriminate between different infecting bacteria in minutes, providing a promising tool for diagnostic and screening applications.

## 1. Introduction

Macrophages are key innate immune cells that are the first line of defense against pathogens, responsible for the phagocytic uptake and killing of bacteria [1] and other pathogens [2,3]. Macrophages also play a major role in the host inflammatory response to bacterial infections, both in terms of the innate [4] and adaptive immune response [5]. Macrophages recognize and phagocytose bacteria, then ultimately recruit the appropriate machinery such as autophagy to eliminate pathogens [6,7]. Opportunistic bacterial pathogens have specific mechanisms to invade macrophages and evade host immune response, including modulating host cell surfaces and releasing proteins to inhibit host immune factors [8,9,10]. The bacteria residing within these host cells can then escape, further propagating infection [11]. These intracellular reservoirs for pathogens exacerbate a wide range of chronic and persistent infections that significantly contribute to patient mortality for diseases including pneumonia [12], chronic osteomyelitis [13], urinary tract infection [14], and lung infections in patients suffering from cystic fibrosis [15]. Therefore, rapid detection and diagnosis of intracellular macrophage infections are important for choosing appropriate and effective treatments [16,17].

The innate immune system has a network of multiprotein complexes that generate biomarkers for bacterial infection recognition [18,19,20]. Current intracellular infection detection methods use these protein biomarkers to detect bacterial infection using reverse transcription-polymerase chain reaction (RT-PCR) [21] or immunoassays [22]. However, there is a complex overlap between the irregular expression of multiple biomarkers presented on macrophages infected by different species of bacteria, resulting in increased complexity in analysis when using biomarker-based approaches [23]. Moreover, it is challenging to identify infections from other bacteria species and develop a generalized diagnostic platform [24].

Array-based sensing offers an alternative to conventional biomarker-based approaches [25,26,27,28,29,30]. This "signature"-based approach enables the detection of phenotypic changes through selective and differential cross-reactive interactions between cells and sensor elements [31,32,33]. These differential interactions result in the generation of unique patterns for each phenotype, which can be further classified for identification. The cross-reactivity of the sensor system provides a hypothesis-free, rapid, and generalized platform for the detection of phenotypic differences [34]. Hypothesis-free cross-reactive sensor platforms have been applied in complex biological systems [35,36,37,38], including proteins [39,40,41], bacteria [42,43,44], and mammalian cells [45,46,47].

**Figure 1:**
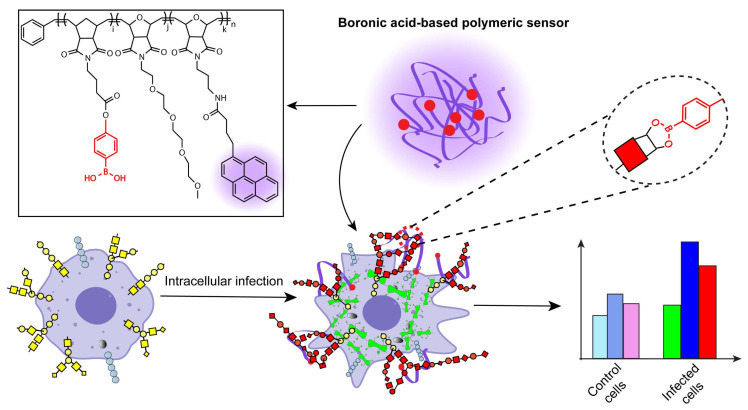
Detection of phenotypic changes in macrophages induced by intracellular bacterial infection using a single fully synthetic, pH-responsive, high-content boronic-acid functionalized polymeric sensor array. The sensor is a random co-polymer, where n is the average monomeric repeats in the polymer, and i, j, and k indicate the ratio of the individual monomeric units in the polymer. The purple tinge represents the fluorescence response from the pyrene and the red spherical dots are representative of the boronic acid functional groups in the polymer. The yellow, red dots and the bluish gray spheres in the cartoon cell represent cell surface glycans. The green cylindrical figures inside the cell represents bacteria.

Cell surface glycosaminoglycans are a prime target for pathogens for initiating infections. Multiple bacteria have developed different mechanisms for manipulating the cell surface glycosaminoglycans at various stages of pathogenesis [48]. Intracellular infections induce widespread alteration of the cell surface glycan composition of immune cells [49,50] including macrophages [51]. Recently, we developed a fully synthetic polymeric sensor array that leveraged the pH-responsiveness and glycan interactions of the boronate functional group to successfully discriminate cell states based on the cell surface glycosylation patterns [52]. We utilized the sensor array to detect changes in the cell surface glycosylation signatures resulting from the induction of malignant phenotype in cancer cells and different drug-induced macrophage polarization states [53]. We, therefore, hypothesized that our pH-responsive synthetic boronic acid-decorated polymer system would be able to detect and identify intracellular infections of macrophages based on the altered glycan composition. This poly(oxanorbornene) (PONI)-based polymer, **PONI-BA-pyrene,** can reversibly and covalently bind with diols present in glycans [54,55], providing the selective recognition required for cell-surface signature identification [56]. This polymeric sensor is pH-responsive driven by the structure and solubility change of boronic acid under different pH, generating a high-content six-channel fluorescent output using three different pH values for sensing. We report here the use of **PONI-BA-pyrene** to detect and identify early stages of bacteria-induced intracellular infection (Figure 1). Using linear discriminant analysis (LDA), discrimination was possible between macrophages infected with low bacterial loads (multiplicity of infection (MOI) 10:1) [57]. The multiplicity of infection is defined here as the ratio of the number of bacteria infecting each macrophage [58]. This rapid detection of intracellular infection provides a new strategy for screening intracellular infections for diagnostic, therapeutic development, and fundamental applications.

## 2. Materials and Methods

### 2.1. Materials

All chemicals and solvents were purchased from Sigma-Aldrich (St. Louis, MO, USA) or Fisher Scientific (Hampton, NH, USA) unless otherwise stated. 1H NMR was recorded on a Bruker ADVANCE 400 machine (Bruker, Billerica, MA, USA). Absorbance and fluorescence were measured using a SpectraMax M2 plate reader (Molecular Devices, San Jose, CA, USA).

### 2.2. Cell Culture

RAW 264.7 cells were purchased from the American Type Culture Collection (ATCC, Manassas, VA, USA) and cultured at 37 °C under a humidified atmosphere containing 5% CO_2_ using standard growth media consisting of high glucose Dulbecco’s Modified Eagle Medium (DMEM) supplemented with 10% fetal bovine serum (FBS). Under the above culture conditions, the cells were sub-cultured approximately once every two days.

### 2.3. Synthesis of PONI-Boronic Acid-Pyrene Polymer

Monomers and polymers were synthesized according to previous reports [59,60]. Detailed synthetic schemes and the corresponding characterization can be found in the Supporting Information.

### 2.4. Infection Model

RAW 264.7 cells (250,000 cells/dish) were plated in confocal dishes overnight. The medium was then replaced with IDRL-12570 red fluorescent protein-expressing methicillin-resistant *Staphylococcus aureus* (MRSA)-containing medium at an MOI of 10:1. The cells were incubated with the medium for 1 h. The cells were then washed and incubated with gentamicin for 30 min to wash extracellular bacteria [61]. Afterward, the cells were incubated with fresh medium for 6 h, and then confocal microscopy was performed to observe the presence of bacteria inside RAW 264.7 cells. Confocal microscopy of RAW 264.7 cells without bacteria treatment was performed as the negative control.

### 2.5. Trypan Blue Exclusion Test of Cell Viability

RAW 264.7 cells (10,000 cells per well) were seeded into a 96-well plate and left overnight. The following day, the medium was replaced with bacteria-containing medium at multiplicities of infection (MOI) of 10:1 or 100:1 and incubated for 1 h. Then, the cells were washed and treated with gentamicin for 30 min to eliminate extracellular bacteria. Subsequently, the cells were incubated with fresh medium for 6 h, followed by a PBS wash. Cells were then treated with 50 µL of trypsin for 10 min to facilitate trypsinization and transferred to 600 µL microcentrifuge tubes for centrifugation at 3000 rpm for 5 min. The supernatant was discarded, and the cells were resuspended in fresh medium. An aliquot of 15 µL cell suspension was mixed with 15 µL of 0.4% trypan blue, and 10 µL of the mixture was loaded into a disposable Countess chamber slide for counting with a Countess Automated Cell Counter (Thermo Fisher Scientific, Waltham, MA, USA) [62].

### 2.6. Sensing Protocol

RAW 264.7 cells (10,000 cells/well) were seeded in a 96-well plate overnight. The medium was then replaced with bacteria-containing medium at an MOI of 10:1. After 1 h incubation of cells with the medium, the cells were washed and incubated with gentamicin for 30 min to wash extracellular bacteria. Afterward, the cells were incubated with fresh medium for 6 h, and then incubated with the polymeric sensor array. After 30 min, fluorescence intensities under 398 nm (pyrene monomer) and 466 nm (pyrene excimer) were recorded using the microplate reader at 25 °C. Finally, six characteristic fluorescent channels were generated from 3 representative phosphate buffers with different pH values (pH 5.8—Monomer, pH 5.8—Excimer, pH 7.4—Monomer, pH 7.4—Excimer, pH 8.2—Monomer, and pH 8.2-Excimer) from a single polymer. The RAW 264.7 cells without bacteria treatment were used as the negative control.

### 2.7. Linear Discriminant Analysis

Linear discriminant analysis (LDA) is a machine learning multivariate technique that identifies a linear combination of features to effectively distinguish between two or more classes of objects [63]. SYSTAT (version 13, Systat Software, Richmond, CA, USA) was employed to generate an LDA plot of the normalized fluorescence intensity (I/I_0_). In SYSTAT, all variables were utilized in complete mode, with a tolerance set to 0.001. The raw fluorescence response patterns were transformed into canonical patterns, maximizing the ratio of between-class variance to within-class variance based on preassigned grouping [64,65].

### 2.8. Identification of Unknown Samples

To identify unknown samples, the fluorescence response patterns of each new case were first converted to canonical scores using the discriminant functions developed from the training data. Subsequently, the Mahalanobis distance to the centroid of each training group was calculated, followed by assessing the probability of cells belonging to the nearest cluster using an appropriate F-distribution for the minimum distance [66].

## 3. Results and Discussion

### 3.1. Synthesis and Characterization of PONI-BA-Pyrene

**Figure 2:**
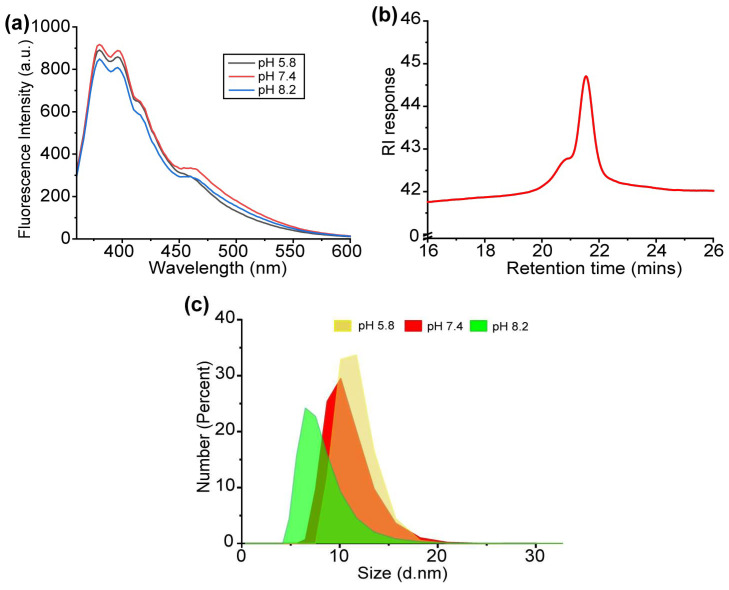
(**a**) Fluorescence spectrum of **PONI-BA-pyrene** sensor at three different pH values. (**b**) GPC trace of Boc-protected **PONI-BA-pyrene** shows the presence of a polymer with Mw = 30 kDa, Mn = 29.6 kDa, and a polydispersity index of 1.01 using polystyrene as the standard, tetrahydrofuran (THF) as the eluent with a flow rate of 1 mL/min. (**c**) The average size of the **PONI-BA-pyrene** sensor array in the three representative pH values was confirmed by a representative DLS spectrum (number).

The sensor array was developed based on a fully synthetic dye-conjugated polymer [67]. The polymer used a poly(oxanorborneneimide) (PONI) random copolymer scaffold for its unique “semi-arthritic” structural properties and ease of modification [68]. The “semi-arthritic” backbone from the PONI polymer provides a good balance between the rigidity and flexibility required to form a highly responsive sensing system. The use of phenylboronic acid on the PONI backbone provides a pH-responsive recognition element that shows a high binding preference to diols present in cell surface glycans [69]. This binding can be transduced by pyrene conjugated to the PONI backbone to provide two-channel fluorescence: pyrene monomer (398 nm) and pyrene excimer (466 nm). Finally, six characteristic fluorescent channels were generated from three representative phosphate buffers with different pH values (pH 5.8—Monomer, pH 5.8—Excimer, pH 7.4—Monomer, pH 7.4—Excimer, pH 8.2—Monomer, and pH 8.2—Excimer) from a single polymer (Figure 2a) [70]. These three pH values were chosen to provide acidic, neutral, and basic environments but ensure the stability of the phosphate buffer. The synthesized polymer **PONI-BA-pyrene** was characterized by gel permeation chromatography (GPC) (Figure 2b) with refractive index (RI) as the detector, with an observed molecular weight of 30 kDa and a polydispersity of 1.01. The hydrodynamic diameter of the polymer was checked under different pH values through dynamic light scattering (DLS) (Malvern Zetasizer Nano ZSP, Malvern Panalytical Inc., Westborough, MA, USA) with observed diameters of 11.3 ± 1.8 nm (pH 5.8), 10.4 ± 2.2 nm (pH 7.4), and 7.7 ± 2.1 nm (pH 8.2) (Figure 2c). The results demonstrated that no substantial aggregation was observed with the change in pH values.

### 3.2. Evaluation of the Glycan Binding Affinity of the Polymeric Sensor

**Figure 3:**
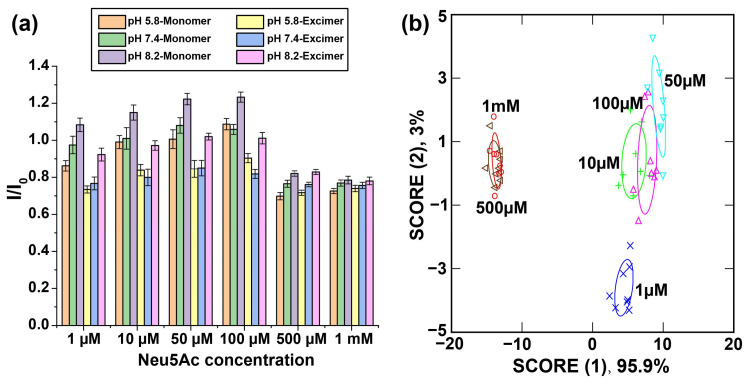
(**a**) Fluorescence response of **PONI-BA-Pyrene** after 30 min incubation with different concentrations of Neu5Ac (n = 8). I/I_0_ is the fluorescence response of the sensors against the analytes normalized to control sensor only. The colors in the bar graph represent the different fluorescent emission channels at different pH values. (**b**) LDA was used to analyze the fluorescence response, and the first two canonical scores were plotted with 95% confidence ellipses.

N-acetylneuraminic acid (Neu5Ac) is the predominant sialic acid found in humans and is presented as the terminal residue in surface-exposed glycans on mammalian cell membranes [71,72]. The aim of detecting Neu5Ac was to use it as a model system to investigate the pH responsiveness and the concentration dependence of the cell surface glycans that modulate the interactions with our sensor. We established the binding of 40 μg L^−1^ **PONI-BA-pyrene** with Neu5Ac under different pH conditions. We chose to work with the physiologically relevant concentration range of Neu5Ac to demonstrate the correlation between changes in Neu5Ac concentrations and sensor response [73,74]. The solutions were incubated for 30 min, and the ratio of the fluorescence intensity of **PONI-BA-pyrene** incubated with Neu5Ac to the fluorescence intensity of **PONI-BA-pyrene** only (I/I_0_) was determined (Figure 3a). The characteristic fluorescent response demonstrated the binding of our sensor with Neu5Ac was pH-responsive, and the polymeric sensor array generated unique fluorescence signatures after incubation with different concentrations of Neu5Ac. The fluorescence pattern was analyzed using linear discriminant analysis (LDA) for dimension reduction (six dimensions to two dimensions) and better quantitation. LDA was chosen for the data analysis as it is a supervised machine learning-based statistical analysis tool that can be trained *a priori*, which becomes particularly useful for predicting unknown outcomes (Figure 3b, Tables S1 and S2) [75].

### 3.3. Validation of the Bacterial Infection Model in Macrophages

**Figure 4:**
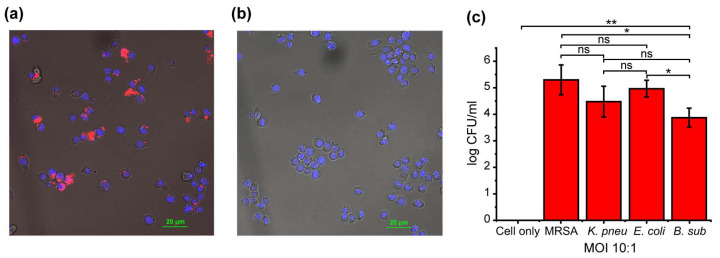
(**a**) Confocal microscopy of RFP-expressing MRSA-infected RAW 264.7 cells (MOI = 10:1). (**b**) Confocal microscopy of RAW 264.7 without treatment as control cells. (**c**) Colony counting of different bacteria-infected RAW 264.7 macrophages. The data shown here are an average of three replicates. Statistical significance was determined by a two-tailed Student’s *t*-test. * = *p* < 0.05, ** = *p* < 0.01, ns = non-significant.

RAW264.7 (murine macrophage) cells were used as representative macrophages, and 250,000 cells were plated in confocal dishes overnight. The medium was then replaced with IDRL-12570 red fluorescent protein-expressing methicillin-resistant *Staphylococcus aureus* (MRSA)-containing medium at an MOI of 10:1. The cells were incubated with the medium for 1 h. The cells were then washed and incubated with gentamicin for 30 min to wash extracellular bacteria. Afterward, the cells were incubated with fresh media for 6 h and then confocal microscopy was performed to observe the presence of bacteria inside RAW 264.7 cells (Figure 4a), and the confocal microscopy of RAW 264.7 without bacteria treatment was also performed as the negative control (Figure 4b). Quantitation of the MOI was obtained using a colony counting assay using 10,000 RAW 264.7 cells. After overnight incubation, the cells were washed and the medium was replaced with the different strains of bacteria (MRSA, *Escherichia coli* (*E. coli*)*, Bacillus subtilis* (*B. sub*)*, Klebsiella pneumoniae* (*K. pneu*)) at MOI 10:1. Colony counting data demonstrated the successful infection of RAW 264.7 cells with the different bacteria (Figure 4c). RAW 264.7 cells infected with *B. sub* showed a slightly lower bacterial load which could be attributed to some *B. sub* suffering exocytosis by the host macrophage [76]. A membrane integrity assay was performed for each of the respective bacteria-infected cells using the Trypan blue exclusion assay. The Trypan blue assay did not show any major difference in the membrane integrity of the macrophages even at a higher MOI of 100:1 (Figure S1), indicating that the infected cells’ membranes remained intact.

### 3.4. Detection and Discrimination between Different Types of Bacteria-Infected Macrophages

The validated intracellular infection model above (MOI 10:1) was used to assess the ability of **PONI-BA-pyrene** to detect intracellular infection. RAW 264.7 cells were infected with the above-mentioned different bacteria species. Two of the bacteria chosen (MRSA, *B. sub*) are Gram-positive and the other two (*E. coli* and *K. pneu*) are Gram-negative. The sensing experiment followed a similar protocol to that of the infection model. In brief, 10,000 RAW 264.7 cells were seeded in a 96-well plate the night before the experiment. The protocol described above was used to establish the infection the following day.

**Figure 5:**
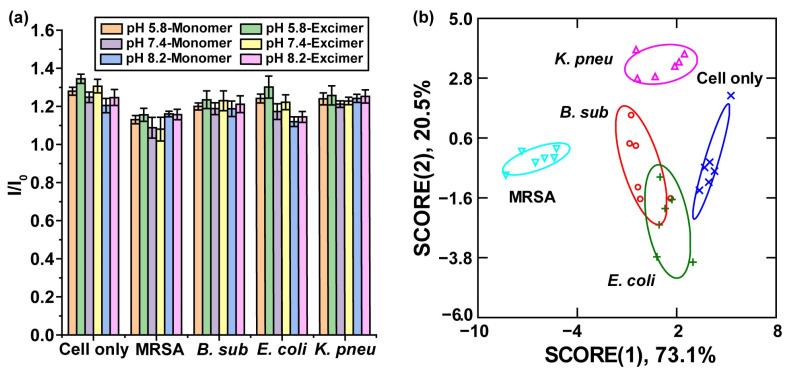
Discrimination between different bacteria-infected RAW 264.7 cell lines showing the sensitivity of the sensor platform to distinguish between the different intracellular infection agents. (**a**) Fluorescence intensities of sensor array after 30 min incubation with each cell line, normalizing against sensor only. Each value is the average of 6 replicates (n = 6). (**b**) Fluorescence patterns were analyzed using LDA and the first two canonical scores were plotted with a 95% confidence ellipse.

After the establishment of the infection, the polymeric sensor was then added to the wells and incubated with the cells for 30 min. The fluorescence was then read out using a microplate reader (Figure 5a). The sensor array generated distinguishable fluorescent signatures for the infected cells with the non-infected along with a substantial difference in fluorescence signature between different bacteria-infected macrophages. LDA was performed on fluorescence data to obtain distinct separate clusters with 90% classification accuracy in cross-validation studies. An overlap was observed between *B. sub-* and *E. coli-* infected cells. The overlap is not surprising, as *B. sub* and *E. coli* have the possibility of employing similar pathways of infection against macrophages (Figure 5b, Table 1 and Table S3) [77,78,79]. The scores generated from the LDA analysis are shown in the axes of Figure 5b. These values visualize the contribution of multiple variables in the sensing protocol that leads to our generated result. To investigate the contribution of pyrene monomer and excimer on the fluorescence response of the sensor toward the infection, we also used LDA to analyze the fluorescence data from monomeric pyrene or excimer (Figure S2). The 73% classification from both pyrene monomer and excimer demonstrated that pyrene monomer and excimer can act simultaneously as recognition sites (Tables S4 and S5), providing a more sensitive tool for the detection of microenvironmental changes [80]. In addition, to validate the sensitivity and accuracy of our sensing platform for identifying macrophages infected by different bacteria, unknown identification was performed, and a 77% correct unknown identification was obtained (Table S6). Overall, the results demonstrate the potential of the **PONI-BA-pyrene** sensing platform for intracellular infection discrimination.

To further evaluate the validity of our sensor system, we proceeded to perform competitive assays for the detection of intracellular infection through cell surface glycan changes. We utilized WGA conjugated with CF555 dye (5 µg mL^−1^) to investigate the impact of intracellular infection on macrophage cell membranes via confocal microscopy. Wheat germ agglutinin (WGA), a lectin known for its affinity towards sialic acids, is commonly employed for the specific labeling of cell membranes [81]. WGA binds to specific sugars among the cell surface glycans and is useful in the diagnosis of glycosylation changes owing to the changes in the specific carbohydrate [82]. The infection was established, and the imaging was performed using the same protocol as used during the validation of the intracellular infection. The obtained micrographs, depicted in Figure S3, revealed no significant difference between the control and bacteria-infected cells, thereby affirming the efficacy of our methodology in discerning subtle variations in glycan composition. Furthermore, we harnessed a sensor array comprising 5 µg mL^−1^ of CF555 and Alexa Fluor 568-conjugated WGAs each to conduct a comparative sensing experiment on multiple bacteria-infected macrophages. The WGAs were added to the infected cells and incubated for 30 min and then the fluorescence was read out using a microplate reader. The results, as shown in Figure S4, demonstrated a modest discrimination rate of 68% utilizing the WGA array (Tables S7 and S8). These results underscore the potential of our pH-responsive polymer sensor array in elucidating intricate alterations in cellular glycan profiles, thereby offering valuable insights into the dynamics of cellular responses to pathogenic invasion.

## 4. Conclusions

In summary, the high-content polymeric BA-based sensor was able to perform a rapid and efficient detection of intracellular bacterial infection of macrophages. The high sensitivity of the **PONI-BA-pyrene** sensor towards the alteration of cell surface glycans allowed for the accurate discrimination of infected and non-infected cells through the difference in their cell surface glycosylation pattern. Furthermore, the sensor array could distinguish between the different strains of bacterial infection. We also performed a comparative study using WGA and demonstrated the superiority of the PONI-BA-pyrene sensor platform in detecting early signs of intracellular bacterial infection. The unique advantages provided by this single polymer-based sensor platform enable the development of tools for studying the effects of bacterial infection on the phenotype of immune cells, providing valuable information for understanding the mechanism of immune evasion by bacteria. Overall, our sensor system provides a multi-purpose utility platform for studying and understanding the effects of bacterial infection on cell surfaces and accelerating the diagnosis and therapeutic discovery for intracellular infection.

## Figures and Tables

**Table 1:** Percentage of accurate classification of RAW 264.7 macrophages infected by different bacteria from Jackknifed analysis. The results show an overall 90% correct classification.

	Cell Only	MRSA	*B. sub*	*E. coli*	*K. pneu*	Correct (%)
**Cell only**	5	0	0	0	1	83
**MRSA**	0	6	0	0	0	100
***B. sub***	1	0	5	0	0	83
***E. coli***	0	0	1	5	0	83
***K. pneu***	0	0	0	0	6	100
**Total**	6	6	6	5	7	**90**

## Data Availability

The original contributions presented in the study are included in the article/Supplementary Material; further inquiries can be directed to the corresponding author/s.

## Supplementary Materials

The following supporting information can be downloaded at: https://www.mdpi.com/article/10.3390/bios14080360/s1. Figure S1: Membrane integrity assay of RAW 264.7 cells; Figure S2: LDA plot of pyrene monomer and excimer peaks calculated separately; Figure S3: Confocal mmicroscopy images of RAW 264.7 cells stained with CF555 conjugated WGA; Figure S4: Fluorescence response and LDA plot of CF555 and AlexaFluor 568 conjugated WGA against infected RAW 264.7 cells. Table S1: Normalized fluorescence responses and LDA output for PONI-BA-pyrene incubating with Neu5Ac; Table S2: Percentage of accurate classification of different concentrations of Neu5Ac incubating with PONI-BA-pyrene from Jackknifed analysis; Table S3: Normalized fluorescence responses and LDA output for RAW 264.7 cells infected by different types of bacteria; Table S4: Percentage of accurate classification of infected RAW 264.7 macrophages using pyrene monomer fluorescence response from Jackknifed analysis; Table S5: Percentage of accurate classification of infected RAW 264.7 macrophages using pyrene excimer fluorescence response from Jackknifed analysis; Table S6: Prediction of RAW 264.7 cells infected by unknown bacteria using training set from Figure 5 and Table S3; Table S7: Normalized fluorescence responses and LDA output for RAW 264.7 cells infected by different types of bacteria when treated with CF555 conjugated WGA and Alexa Fluor 568 conjugated WGA; Table S8: Percentage of accurate classification of RAW 264.7 macrophages infected by different bacteria from Jackknifed analysis.
