# Supplementary material for: Nanosensor-Enabled Detection and Identification of Intracellular Bacterial Infections in Macrophages
Source: Biosensors (Basel). 2024 Jul 25;14(8):360. doi: 10.3390/bios14080360 (PMC11352223; doi:10.3390/bios14080360)
Supplement: Supplementary file 1 [file biosensors-14-00360-s001.zip › biosensors-3094259-supplementary.pdf]

biosensors

Supplementary Materials

Nanosensor-Enabled Detection and Identification of Intracellular Bacterial Infections in Macrophages

Aritra Nath Chattopadhyay +, Mingdi Jiang +, Jessa Marie V. Makabenta, Jungmi Park, Yingying Geng and Vincent Rotello *

Department of Chemistry, University of Massachusetts Amherst, 710 North Pleasant Street, Amherst, MA 01003, USA
* Correspondence: rotello@chem.umass.edu
+ These authors contributed equally to this work.

1. Synthesis of PONI-boronic acid-pyrene polymer

The PONI-boronic acid-pyrene polymer was synthesized using a previously reported procedure [i].

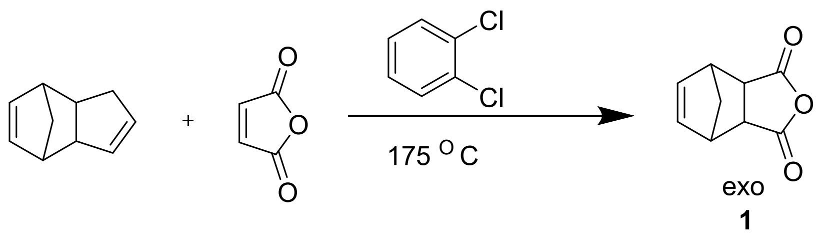

Synthesis of **1**

Maleic anhydride (30 g, 370 mmol, 2.0 eq) was refluxed in 60 mL of o-dichlorobenzene in a 250 mL round bottom flask. Dicyclopentadiene (24.5 g, 185 mmol, 1.0 eq) melted in 50 °C hot water for 10 minutes was then transferred to the reaction flask dropwise over 15 minutes, and the reaction was run at reflux for another 1.5 hours. After cooling down at room temperature for 2 hours, the flask was placed in the refrigerator for further cooling down. After 12 hours, the resulting crystalline solid was filtered off in vacuo. Multiple recrystallizations in boiling monochlorobenzene resulted in the isolation of the desired compound **1** (45% yield) with a high purity. ^1H NMR (400 MHz, $CDCl_3$) 6.34 (s, 2H), 3.45 (s, 2H), 3.01 (s, 2H), 1.79 (d, J=11Hz, 1H), 1.57 (d, J=11Hz, 1H).

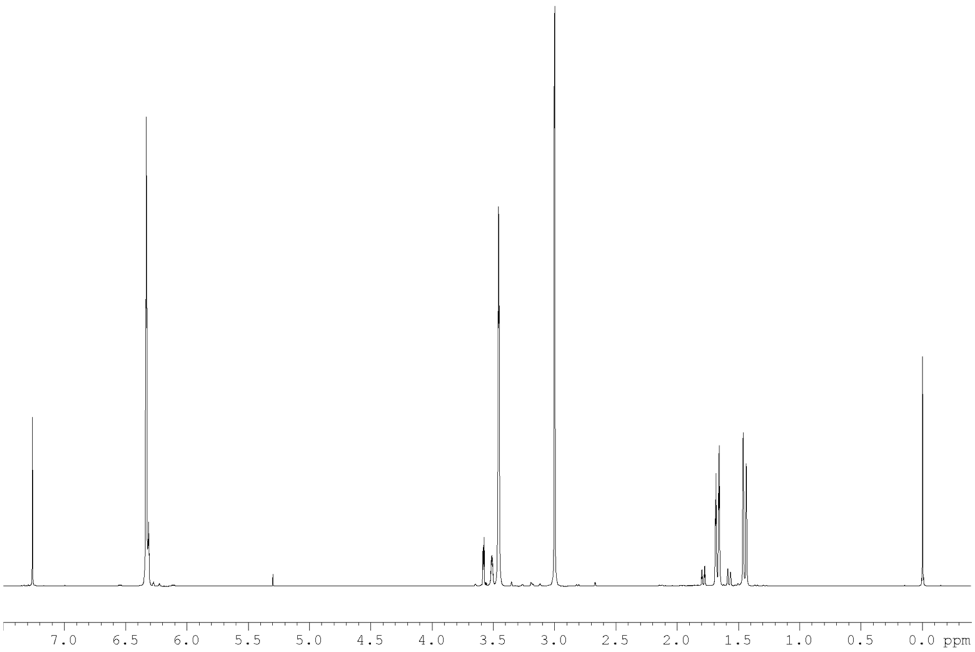

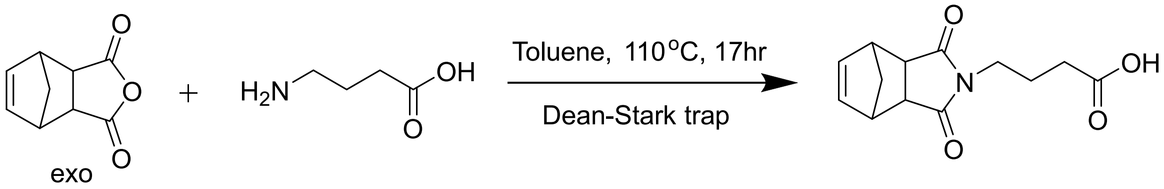

1 **2**

Synthesis of **2**

1 (1 g, 6.09 mmol, 1.0 eq) and 4-aminobutyric acid (0.69 g, 6.70 mmol, 1.1 eq) were added to a 250 mL round-bottom flask equipped with a stir bar and 50 mL of toluene. The reaction mixture was connected to a Dean–Stark trap, heated to 110 °C and run overnight. Then, the reaction mixture was cooled down to room temperature, washed with 1M HCl (3x, 30 mL), water (3x, 30 mL), and brine (1x, 30 mL). The organic layer was dried with sodium sulfate, filtered, and rotavaped. Column chromatography was performed to yield **2** as a white solid (76% yield). ^{1}H NMR (400 MHz, $CDCl_3$) 6.32 (s, 2H), 3.55 (t, 2H), 3.28 (s, 2H), 2.70 (s, 2H), 2.39 (t, 2H), 1.92 (q, 2H), 1.53 (m, 1H), 1.21 (m, 1H).

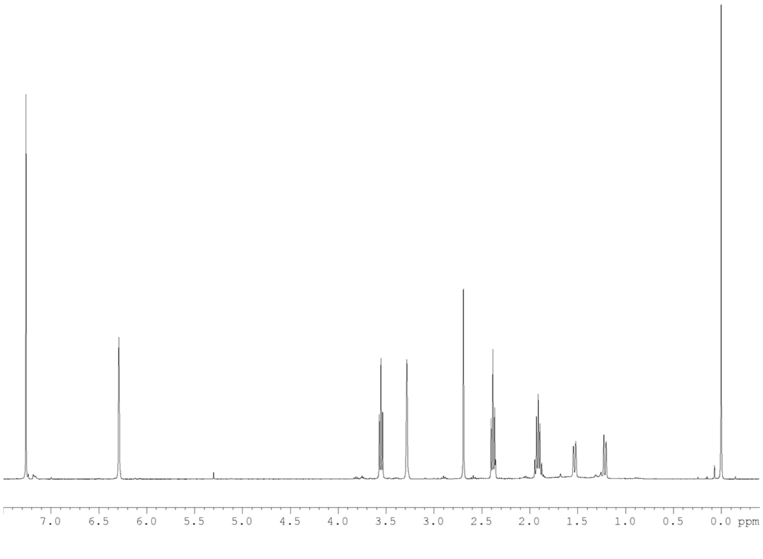

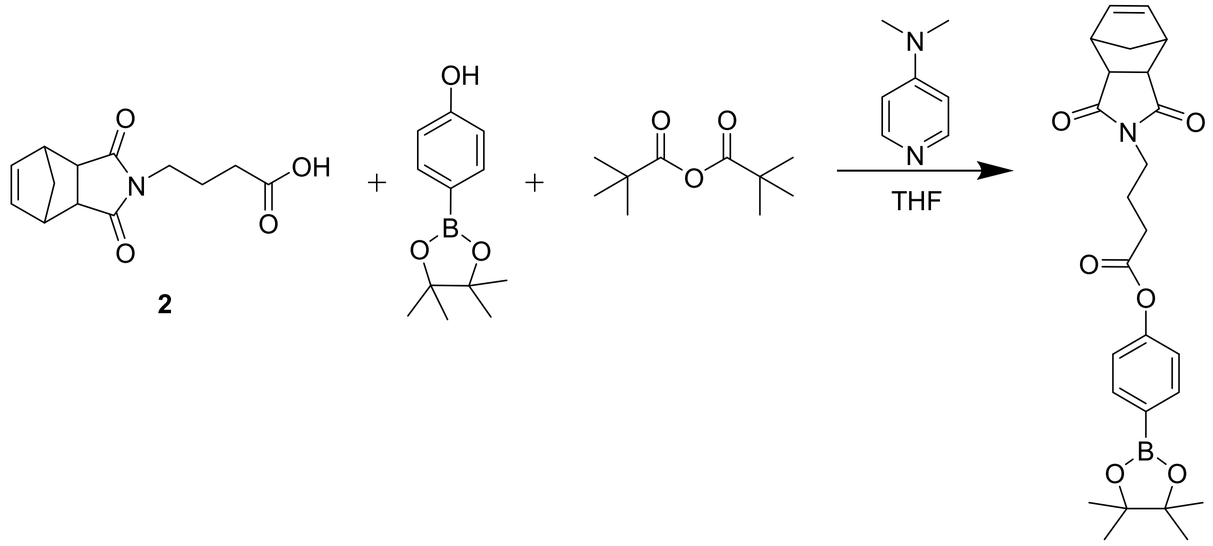

2

3

Synthesis of **3**

2 (1.1 g, 4.4 mmol, 1.1 eq), 4-hydroxyphenylboronic acid pinacol ester (0.9 g, 4.0 mmol, 1.0 eq), and 4-dimethylaminopyridine were mixed with dry tetrahydrofuran (THF) and refluxed in a 250 ml round-bottom flask. The mixture was stirred until everything was dissolved and clear. Then, pivalic anhydride (0.8 g, 4.4 mmol, 1.1 eq) was added to the reaction mixture, and the reaction was kept stirring at reflux condition. After 24 hours, 5 mL of water was added and stirred for 2 hours. Dichloromethane (DCM) was then added to the reaction mixture, washed with saturated sodium bicarbonate (3x) and brine (1x), and dried over $MgSO_4$. After that, the mixture was filtered and concentrated on a rotary evaporator. Column chromatography was done in silica with 33% ethyl acetate in hexanes, which resulted in a white solid with 68% yield. ^1H NMR (400 MHz, $CDCl_3$) 7.82 (d, 2H), 7.10 (d, 2H), 6.27 (s, 2H), 3.61 (t, 2H), 3.28 (s, 2H), 2.69 (s, 2H), 2.58 (t, 2H), 2.00 (m, 2H), 1.55 (m, 2H), 1.33 (s, 12H).

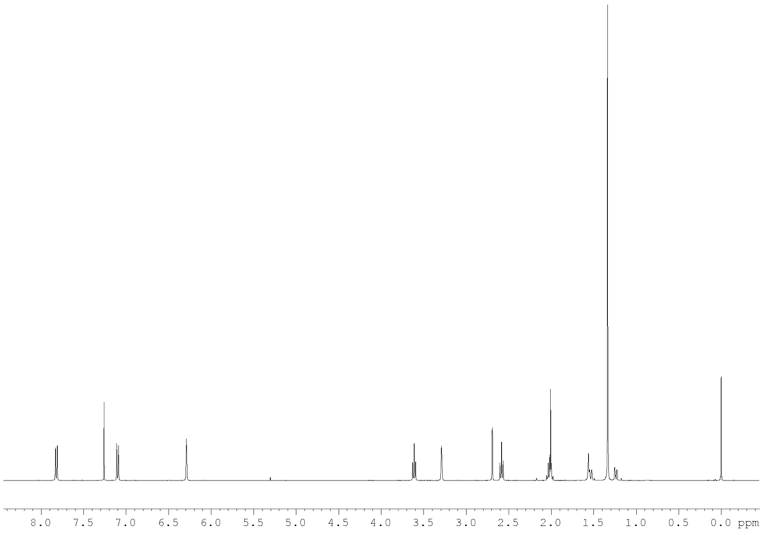

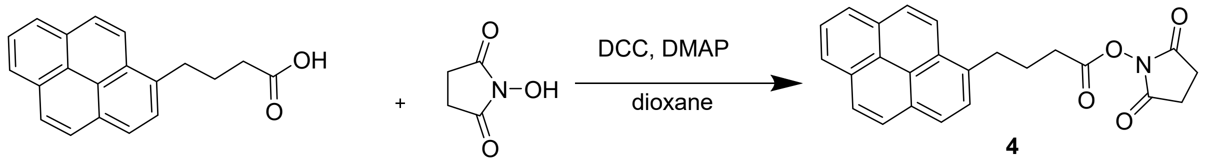

Synthesis of **4**

Pyrene butyric acid (2.0 g, 4.4 mmol, 1.1 eq) and N-hydroxysuccinimide (0.9 g, 4.0 mmol, 1.0 eq) were mixed and dissolved in dioxane in an ice bath. Dicyclohexylcarbodiimide was added to the mixture at 0 °C and the reaction mixture was stirred for 15 minutes. 4-dimethylaminopyridine was next added to the mixture at 0 °C and stirred in the ice bath. After 1 hour, the ice bath was removed, and the reaction was left to run overnight. After 18 hours, the mixture was concentrated using a rotary evaporator. Column chromatography in silica with 50% ethyl acetate in hexanes yielded **4** as a yellowish-white solid with 37% yield. ^{1}H NMR (400 MHz, $CDCl_3$) 8.30 (d, 1H), 8.15 (m, 4H), 8.00 (m, 3H), 7.88 (d, 1H), 3.45 (t, 2H), 2.90 (s, 4H), 2.84 (t, 2H), 2.30 (p, 2H).

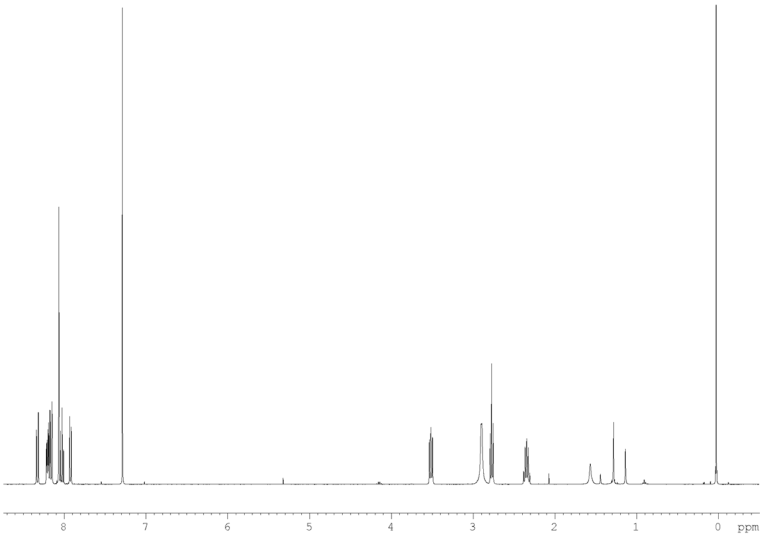

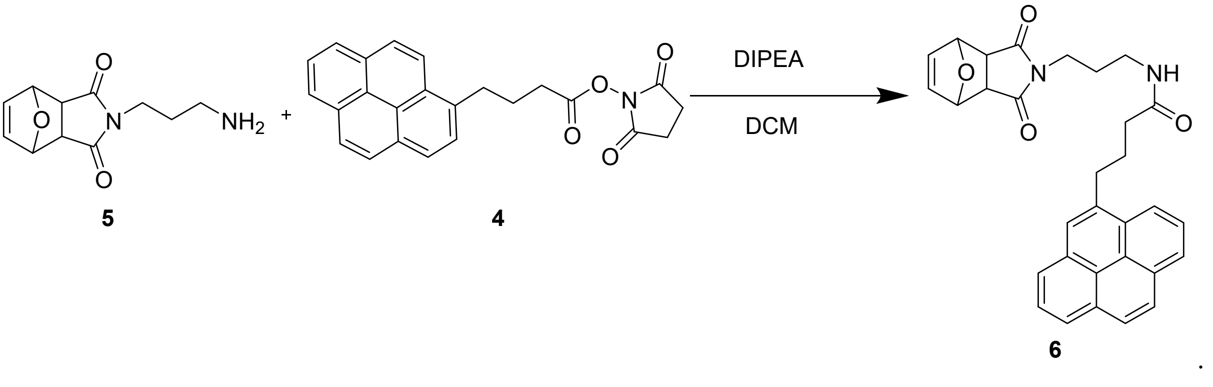

Synthesis of **6**

Monomer **5** was synthesized following previous reports [ii]. **5** (0.60 g, 1.78 mmol, 1.0 eq) was added to a 250 mL round-bottom flask equipped with a stir bar under N_2 atmosphere at room temperature. Diisopropylethylamine (0.69 g, 5.35 mmol, 3.0 eq) and DCM were added to the flask under N_2 purging, and the mixture was stirred. **4** (0.69 g, 1.78 mmol, 1.0 eq) was slowly added to the reaction, and the mixture was stirred for 4 hours at room temperature. Column chromatography was performed in silica in pure ethyl acetate to yield the product as a slight yellow solid (63% yield). ^{1}H NMR (400 MHz,

$CDCl_3$) 8.34 (d, 1H), 8.18 (m, 4H), 8.00 (m, 3H), 7.88 (d, 1H), 6.48 (s, 2H), 6.17 (s, 1H), 5.21 (s, 2H), 3.54 (t, 2H), 3.42 (t, 2H), 3.18 (m, 2H), 2.39 (t, 2H), 2.25 (m, 2H), 1.76 (m, 2H), 1.60 (s, 1H).

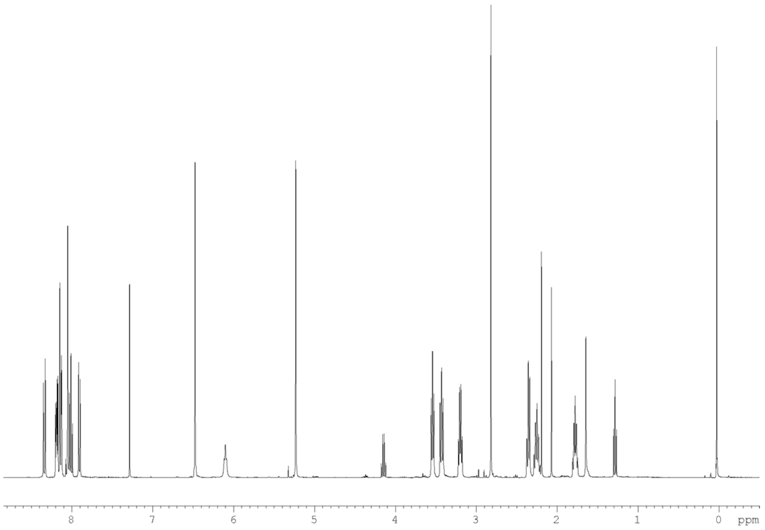

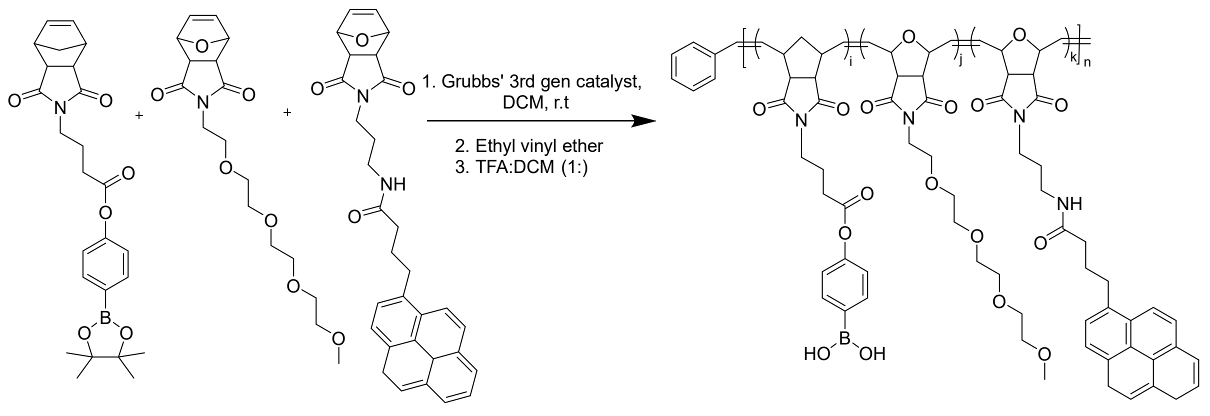

3 **7** **6** **PONI-BA-pyrene**

Polymer synthesis scheme:

Monomer **7** was also synthesized following previous reports [1]. Then, **3** (50 mg, 0.10 mmol, 2.0 eq), **7** (126 mg, 0.33 mmol, 7.0 eq), **6** (25 mg, 0.05 mmol, 1.0 eq), and 5 mL of DCM were added to a 15 mL pear-shaped air-free flask equipped with a stir bar. In a separate 10 mL pear-shaped air-free flask, Grubbs' 3rd generation catalyst (10.0 mg, 0.012 mmol) and 1 mL DCM were added. Both flasks were sealed with septa and attached to a Schlenk nitrogen/vacuum line, and they were freeze-pump-thawed three times. After thawing, Grubbs' 3rd generation catalyst was removed via syringe and quickly added to the flask containing **3, 7,** and **6** and allowed to react for 90 minutes. After that, 300 μL of ethyl vinyl ether was added and stirred for 20 minutes. After 1:1 dilution, the mixture was precipitated into a heavily stirred solution of a 1:1 mixture of ethyl ether and hexane. The precipitated polymer was filtered and dissolved into THF. The polymer was precipitated again into the same mixture solvent and filtered. 1H NMR (400MHz, CDCl3) 8.37 (m, 2H), 8.23 (m, 6H), 8.11 (s, 4H), 8.02 (s, 3H), 7.93 (m, 2H), 7.81 (d, 6H), 7.7 (s, 1H), 7.05 (s, 6H), 5.93 (s, 12H), 5.70 (s, 19H), 4.84 (s, 12H), 4.37 (s, 12H), 3.45 (d, 280H), 3.20 (s, 38H), 3.07 (s, 11H), 2.54 (m, 6H), 2.22 (s, 3H), 2.00 (s, 4H), 1.83 (s, 5H), 1.63 (s, 3H), 1.28 (s, 7H). After filtration, the residue was dissolved in 5 mL of DCM and 5 mL of trifluoroacetic acid, and methyl boronic acid (5.0 eq) was added to the reaction mixture and left to run overnight. Afterward, the solvent was completely evaporated and washed with hexane twice and dissolved into a minimal amount of water. Then, the polymers were added to 10,000

MWCO dialysis membranes and stirred for three days in Milli Q water, changing the water periodically. The polymers were filtered through PES syringe filters and freeze-dried to yield **PONI-BA-pyrene.** The polymer was also characterized by GPC (gel permeation chromatography) in THF. The MW was ~30,000 and the PDI (polydispersity index) was 1.01. The MW was determined by considering the shoulder peak of the GPC chromatograph in the calculations.

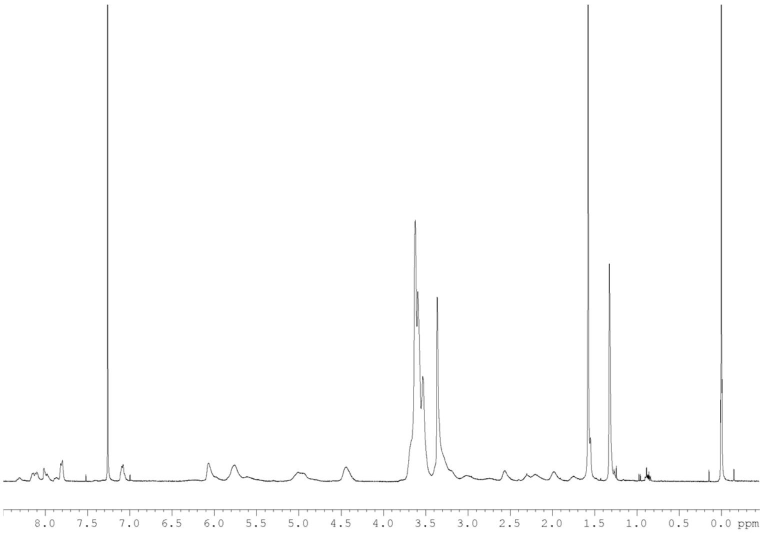

2. Trypan blue exclusion assay

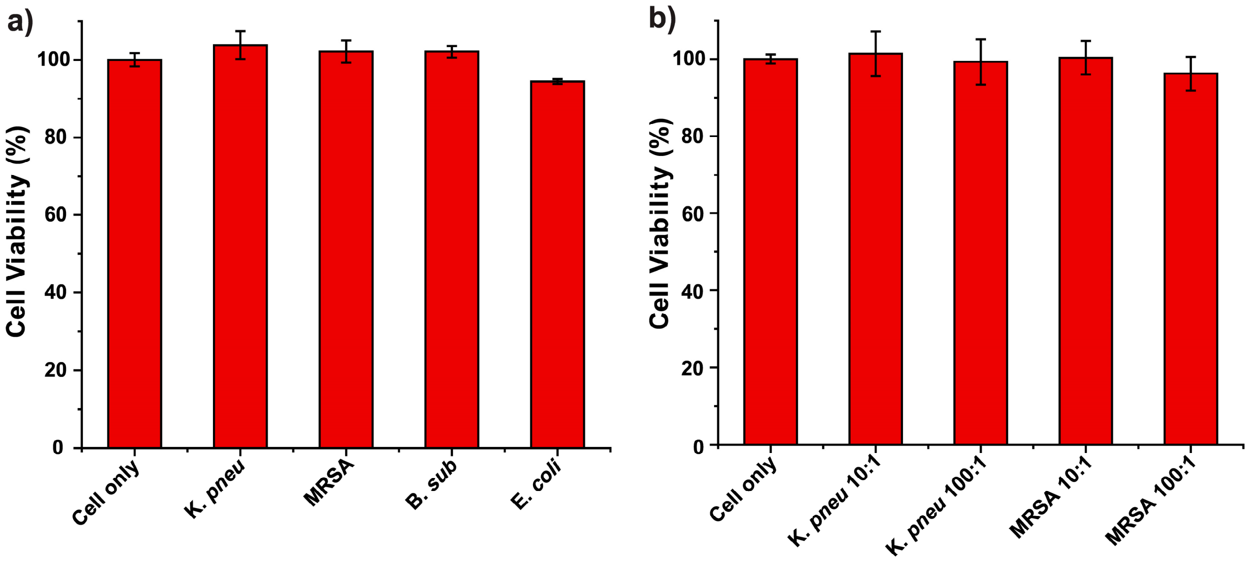

Figure S1. (a) Membrane integrity assay of RAW 264.7 cells treated with four respective bacteria at an MOI 10:1 using trypan blue exclusion assay. (b) Membrane integrity assay of RAW 264.7 cells with different ratios of bacteria including MOIs of 10:1 as well as 100:1.

3. Sensing data

3.1. Sensing data for fluorescence response of PONI-BA-pyrene incubating with different concentrations of N-acetylneuraminic acid (Neu5Ac)

Table S1. Normalized fluorescence responses and LDA output for PONI-BA-pyrene incubating with Neu5Ac. Score (1) and score (2) correspond to Figure 3 in the main text.

**Sample name**	**I/I0**						**LDA output**	
	**pH 5.8-Monomer**	**pH 5.8-Excimer**	**pH 7.4-Monomer**	**pH 7.4-Excimer**	**pH 8.2-Monomer**	**pH 8.2-Excimer**	**Score (1)**	**Score (2)**
**1 µM**	1.213	1.017	1.293	1.225	1.006	1.301	-0.683	3.384
**1 µM**	1.208	1.180	1.126	1.319	1.212	1.155	-1.265	0.679
**1 µM**	1.016	1.156	1.336	1.234	1.179	1.417	-0.138	2.289
**1 µM**	1.155	1.241	1.281	1.257	1.240	1.340	-0.358	1.864
**1 µM**	0.918	1.178	1.104	0.991	1.236	1.139	-1.862	-0.621
**1 µM**	1.114	1.289	1.313	1.072	1.266	1.330	-0.596	2.025
**1 µM**	0.980	0.894	1.012	1.067	0.949	1.057	-2.644	-0.088
**10 µM**	1.070	1.342	1.172	1.167	1.349	1.202	-1.529	0.195
**10 µM**	1.173	1.169	0.982	1.170	1.172	1.060	-2.311	-1.065
**10 µM**	1.013	1.199	1.335	1.113	1.220	1.369	-0.339	2.194
**10 µM**	1.118	1.194	1.040	1.296	1.174	1.109	-2.739	-0.248
**10 µM**	1.086	0.990	0.984	1.156	1.060	1.007	-2.376	-0.524
**10 µM**	1.020	0.933	1.100	1.311	0.958	1.203	-2.108	0.921
**10 µM**	0.940	0.986	0.930	1.057	1.013	1.004	-3.534	-1.367
**10 µM**	0.831	1.033	1.016	0.916	1.125	1.039	-2.595	-1.252
**50 µM**	0.973	1.064	1.089	1.035	1.105	1.123	-2.144	0.007
**50 µM**	1.081	0.931	1.011	1.051	0.940	1.041	-2.841	0.235
**50 µM**	1.101	1.169	1.039	1.187	1.157	1.088	-2.704	-0.268
**50 µM**	1.073	1.037	0.978	1.010	1.025	1.003	-3.296	-0.474
**50 µM**	0.976	1.133	1.081	1.112	1.061	1.089	-4.096	0.731
**50 µM**	0.885	1.106	0.980	0.911	1.116	0.989	-3.823	-1.201
**50 µM**	0.884	1.036	0.964	1.028	1.171	0.981	-2.387	-1.818
**50 µM**	0.904	0.900	0.918	0.966	0.941	0.932	-4.015	-1.026
**100 µM**	1.261	1.226	1.240	1.419	1.495	1.377	4.015	-0.541
**100 µM**	1.284	1.261	1.218	1.347	1.458	1.311	2.737	-0.216
**100 µM**	1.196	1.268	1.293	1.370	1.544	1.462	4.598	-0.586
**100 µM**	1.214	1.397	1.278	1.292	1.563	1.423	3.079	-0.491
**100 µM**	1.294	1.335	1.270	1.462	1.526	1.364	2.953	0.210
**100 µM**	1.172	1.298	1.243	1.233	1.421	1.299	1.251	0.311
**100 µM**	1.169	1.321	1.350	1.208	1.352	1.464	1.313	1.579
**100 µM**	1.059	1.211	1.281	1.147	1.295	1.303	0.222	1.317
**500 µM**	1.198	1.287	1.243	1.277	1.513	1.297	2.739	-0.282
**500 µM**	1.143	1.269	1.227	1.219	1.538	1.372	3.792	-1.372
**500 µM**	1.132	1.342	1.297	1.258	1.658	1.339	4.090	-0.672
**500 µM**	1.205	1.382	1.295	1.330	1.591	1.423	3.540	-0.405
**500 µM**	1.257	1.258	1.327	1.343	1.462	1.410	3.472	0.855
**500 µM**	1.164	1.253	1.275	1.408	1.401	1.408	2.103	0.462
**500 µM**	1.191	1.227	1.233	1.284	1.333	1.404	1.835	0.080
**500 µM**	1.104	1.192	1.214	1.193	1.369	1.277	1.478	-0.063
**1 mM**	1.132	1.092	1.154	1.206	1.212	1.205	0.138	0.318
**1 mM**	1.063	1.108	1.149	1.076	1.243	1.204	0.246	-0.222
**1 mM**	1.044	1.232	1.222	1.243	1.360	1.254	0.238	0.385
**1 mM**	1.181	1.254	1.163	1.139	1.448	1.261	2.164	-1.208
**1 mM**	1.171	1.229	1.203	1.268	1.329	1.317	0.957	0.116
**1 mM**	1.0291	1.195	1.101	0.991	1.401	1.170	1.072	-1.884
**1 mM**	1.081	1.223	1.152	1.131	1.363	1.237	0.687	-0.817
**1 mM**	1.107	1.241	1.145	1.201	1.422	1.280	1.662	-1.446

Table S2. Percentage of accurate classification of different concentrations of Neu5Ac incubating with PONI-BA-pyrene from Jackknifed analysis. The results show an overall 34% correct classification.

	**1 mM**	**1 µM**	**10 µM**	**100 µM**	**50 µM**	**500 µM**	**Correct (%)**
**1 mM**	5	2	0	1	0	0	63

**1 μM**	0	4	2	0	1	0	57
**10 μM**	0	3	1	0	4	0	13
**100 μM**	1	2	0	0	0	5	0
**50 μM**	0	0	4	0	4	0	50
**500 μM**	1	0	0	5	0	2	25
**Total**	7	11	7	6	9	7	**34**

3.2. Sensing data for discrimination of RAW 264.7 infected by different bacteria

Table S3. Normalized fluorescence responses and LDA output for RAW 264.7 cells infected by different types of bacteria. Score (1) and score (2) correspond to Figure 5 in the main text.

**Sample name**	**I/I_0**						**LDA output**	
	**pH 5.8-Monomer**	**pH 5.8-Excimer**	**pH 7.4-Monomer**	**pH 7.4-Excimer**	**pH 8.2-Monomer**	**pH 8.2-Excimer**	**Score (1)**	**Score (2)**
**Cell only**	1.250	1.305	1.289	1.362	1.193	1.234	3.684	-0.485
**Cell only**	1.281	1.354	1.244	1.311	1.210	1.253	4.283	-0.619
**Cell only**	1.276	1.355	1.247	1.296	1.210	1.277	3.372	-1.325
**Cell only**	1.315	1.372	1.221	1.265	1.271	1.306	5.273	2.160
**Cell only**	1.271	1.327	1.217	1.275	1.182	1.222	3.933	-1.025
**Cell only**	1.285	1.355	1.268	1.328	1.160	1.187	3.992	-0.288
**MRSA**	1.136	1.186	1.117	1.119	1.178	1.181	1.885	3.242
**MRSA**	1.162	1.200	1.145	1.129	1.172	1.190	2.138	3.401
**MRSA**	1.134	1.146	1.080	1.078	1.169	1.168	2.467	3.695
**MRSA**	1.137	1.164	1.114	1.110	1.147	1.143	0.699	2.860
**MRSA**	1.115	1.132	0.989	0.959	1.152	1.142	-0.453	3.849
**MRSA**	1.098	1.107	1.084	1.089	1.144	1.117	-0.377	2.796
B. sub	1.210	1.256	1.158	1.185	1.233	1.261	-6.530	-0.304
B. sub	1.195	1.222	1.163	1.200	1.156	1.187	-5.258	0.205
B. sub	1.233	1.318	1.195	1.235	1.188	1.221	-5.395	-0.129
B. sub	1.192	1.222	1.239	1.324	1.139	1.153	-5.978	-0.146
B. sub	1.189	1.190	1.166	1.189	1.170	1.185	-8.318	-0.770
B. sub	1.182	1.200	1.207	1.244	1.239	1.260	-7.355	0.107
E. coli	1.247	1.291	1.124	1.178	1.110	1.150	-0.815	0.381
E. coli	1.255	1.326	1.234	1.280	1.130	1.164	-0.176	-1.657
E. coli	1.234	1.286	1.209	1.259	1.098	1.115	-0.314	-1.223
E. coli	1.226	1.262	1.165	1.203	1.164	1.186	1.662	-1.637
E. coli	1.276	1.403	1.145	1.204	1.097	1.112	-0.438	0.301
E. coli	1.209	1.240	1.159	1.207	1.117	1.142	-0.724	1.425
K. pneu	1.262	1.301	1.210	1.238	1.251	1.258	2.982	-3.967
K. pneu	1.253	1.272	1.231	1.252	1.255	1.274	1.714	-1.660
K. pneu	1.278	1.314	1.219	1.240	1.252	1.261	1.299	-1.993
K. pneu	1.236	1.258	1.218	1.225	1.263	1.298	1.004	-0.834
K. pneu	1.214	1.216	1.210	1.218	1.221	1.215	0.804	-3.770
K. pneu	1.194	1.183	1.180	1.197	1.211	1.208	0.941	-2.592

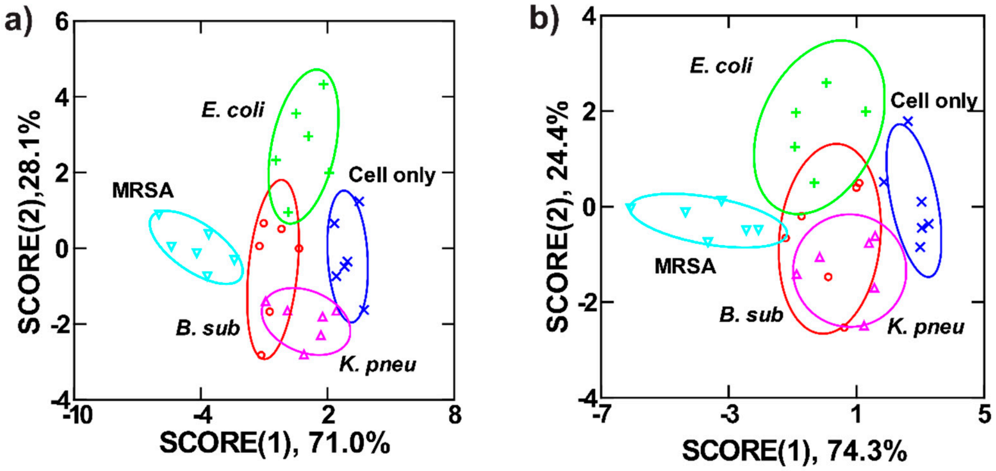

Figure S2. (a) LDA plot of pyrene monomer fluorescence response and the first two canonical scores were plotted with 95% confidence ellipses. (b) LDA plot of pyrene excimer fluorescence response and the first two canonical scores were plotted with 95% confidence ellipses.

Table S4. Percentage of accurate classification of infected RAW 264.7 macrophages using pyrene monomer fluorescence response from Jackknifed analysis. The results show an overall 73% correct classification.

	**Cell only**	**MRSA**	***B. sub***	***E. coli***	***K. pneu***	**Correct (%)**
**Cell only**	5	0	0	0	1	83
**MRSA**	0	5	1	0	0	83
B. sub	0	0	4	0	2	67
E. coli	0	0	1	5	0	83
K. pneu	1	0	2	0	3	50
**Total**	6	5	8	5	6	**73**

Table S5. Percentage of accurate classification of infected RAW 264.7 macrophages using pyrene excimer fluorescence response from Jackknifed analysis. The results show an overall 73% correct classification.

	**Cell only**	**MRSA**	***B. sub***	***E. coli***	***K. pneu***	**Correct (%)**
**Cell only**	6	0	0	0	0	100
**MRSA**	0	6	0	0	0	100
B. sub	1	0	2	1	2	33
E. coli	0	0	1	5	0	83
K. pneu	1	0	2	0	3	50
**Total**	8	6	5	6	5	**73**

Table S6. Prediction of RAW 264.7 cells infected by unknown bacteria using training set from Figure 5 and Table S3. The results show an overall 77% correct unknown identification, but the correct unknown identification is 83% when *E. coli and B. sub* are regarded as one group.

**Unknown sample #**	**I/I0**						**True ID**	**Identified as**	**Correct prediction**
	**pH 5.8-Monomer**	**pH 5.8-Excimer**	**pH 7.4-Monomer**	**pH 7.4-Excimer**	**pH 8.2-Monomer**	**pH 8.2-Excimer**			
**1**	1.279	1.318	1.225	1.274	1.187	1.217	Cell only	Cell only	Yes
**2**	1.310	1.384	1.235	1.264	1.234	1.272	Cell only	Cell only	Yes

**3**	1.323	1.353	1.240	1.274	1.214	1.224	Cell only	*K. pneu*	No
**4**	1.281	1.332	1.213	1.263	1.214	1.237	Cell only	Cell only	Yes
**5**	1.273	1.315	1.218	1.258	1.189	1.209	Cell only	Cell only	Yes
**6**	1.286	1.325	1.223	1.250	1.205	1.206	Cell only	*K. pneu*	No
**7**	1.224	1.231	1.210	1.220	1.222	1.242	*K. pneu*	*K. pneu*	Yes
**8**	1.232	1.262	1.234	1.250	1.221	1.244	*K. pneu*	*K. pneu*	Yes
**9**	1.256	1.280	1.243	1.266	1.235	1.273	*K. pneu*	*K. pneu*	Yes
**10**	1.233	1.283	1.228	1.244	1.249	1.258	*K. pneu*	*K. pneu*	Yes
**11**	1.222	1.221	1.224	1.219	1.218	1.235	*K. pneu*	*K. pneu*	Yes
**12**	1.169	1.161	1.159	1.163	1.196	1.195	*K. pneu*	*K. pneu*	Yes
**13**	1.117	1.158	1.105	1.109	1.147	1.165	MRSA	MRSA	Yes
**14**	1.121	1.124	1.141	1.126	1.190	1.201	MRSA	MRSA	Yes
**15**	1.112	1.119	1.094	1.100	1.179	1.187	MRSA	MRSA	Yes
**16**	1.178	1.249	1.070	1.073	1.134	1.130	MRSA	MRSA	Yes
**17**	1.116	1.129	1.025	1.005	1.130	1.106	MRSA	MRSA	Yes
**18**	1.111	1.127	1.014	0.991	1.132	1.130	MRSA	MRSA	Yes
**19**	1.204	1.325	1.149	1.239	1.140	1.193	*B. sub*	*B. sub*	Yes
**20**	1.204	1.314	1.158	1.249	1.114	1.179	*B. sub*	*B. sub*	Yes
**21**	1.212	1.331	1.121	1.187	1.123	1.171	*B. sub*	*B. sub*	Yes
**22**	1.181	1.287	1.124	1.191	1.122	1.145	*B. sub*	*B. sub*	Yes
**23**	1.183	1.276	1.202	1.280	1.148	1.184	*B. sub*	*B. sub*	Yes
**24**	1.190	1.278	1.185	1.249	1.178	1.208	*B. sub*	*B. sub*	Yes
**25**	1.294	1.412	1.105	1.181	1.087	1.139	*E. coli*	*E. coli*	Yes
**26**	1.192	1.319	1.108	1.163	1.122	1.192	*E. coli*	*B. sub*	No
**27**	1.172	1.283	1.061	1.115	1.086	1.143	*E. coli*	*B. sub*	No
**28**	1.122	1.215	0.903	0.913	1.066	1.103	*E. coli*	MRSA	No
**29**	1.151	1.233	1.053	1.095	1.100	1.145	*E. coli*	MRSA	No
**30**	1.155	1.261	1.125	1.167	1.129	1.179	*E. coli*	MRSA	No

4. Confocal microscopy and sensing data with wheat germ agglutinin (WGA) control

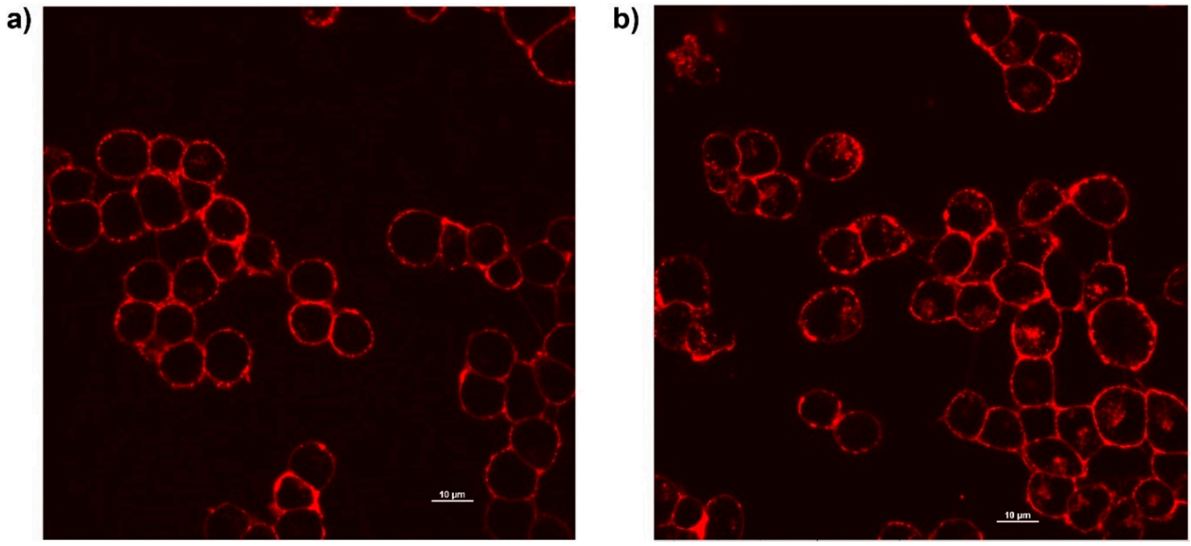

Figure S3. (a) Representative confocal microscopy images of RAW 264.7 cells only as control stained with CF555 conjugated WGA. (b) Representative confocal microscopy image of MRSA-infected RAW 264.7 cells (MOI 10:1) stained with CF555 conjugated WGA. Scale bar: 10 µm.

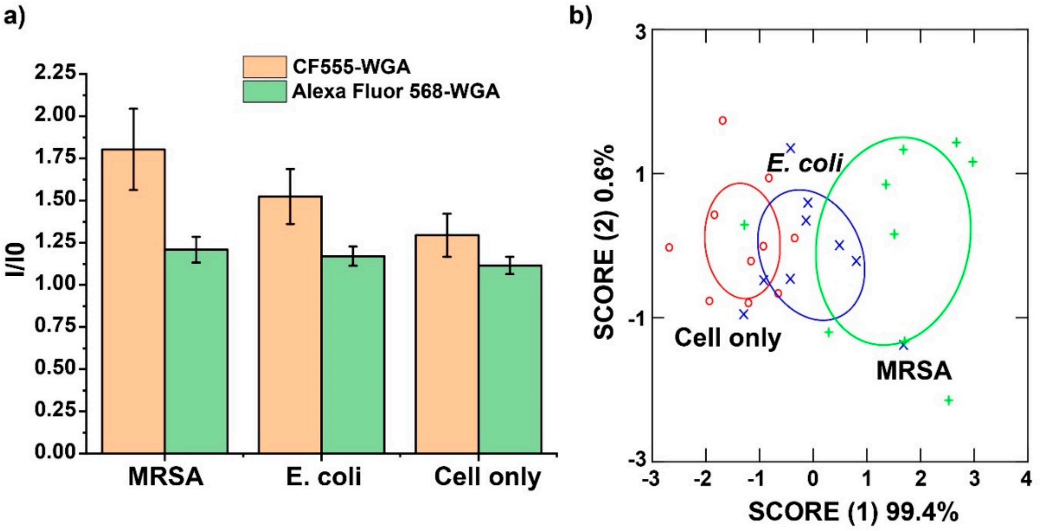

Figure S4. (a) Fluorescence response of **CF555-WGA** and **Alex Fluor 568-WGA** after 30 min incubation with different bacteria-infected cells, normalized to sensor only. Each value is the average of 9 replicates (n=9). (b) LDA was used to analyze the fluorescence response and the first two canonical scores were plotted with 95% confidence ellipses.

Table S7. Normalized fluorescence responses and LDA output for RAW 264.7 cells infected by different types of bacteria when treated with CF555 conjugated WGA and Alexa Fluor 568 conjugated WGA. Score (1) and score (2) correspond to Figure S4.

**Sample Name**	**I/I_0**		**LDA Output**	
	**CF555-WGA**	**Alexa Fluor 568-WGA**	**Score (1)**	**Score (2)**
**Cell only**	1.248	1.011	-1.676	1.728
**Cell only**	1.306	1.168	-1.196	-0.804
**Cell only**	1.395	1.084	-0.814	0.93
**Cell only**	1.409	1.179	-0.64	-0.673
**Cell only**	1.473	1.147	-0.334	0.095
**Cell only**	1.322	1.137	-1.148	-0.223
**Cell only**	1.175	1.142	-1.924	-0.777
**Cell only**	1.048	1.075	-2.672	-0.034
**Cell only**	1.205	1.078	-1.83	0.419
**Cell only**	1.366	1.133	-0.918	-0.016
E. coli	1.822	1.297	1.686	-1.378
E. coli	1.471	1.074	-0.42	1.352
E. coli	1.45	1.175	-0.425	-0.461
E. coli	1.512	1.14	-0.132	0.349
E. coli	1.676	1.203	0.807	-0.214
E. coli	1.287	1.173	-1.294	-0.953
E. coli	1.521	1.127	-0.098	0.597
E. coli	1.36	1.159	-0.92	-0.481
E. coli	1.621	1.18	0.491	0.006
**MRSA**	1.966	1.368	2.529	2.148
**MRSA**	1.304	1.104	1.278	0.288
**MRSA**	1.826	1.295	1.707	1.331
**MRSA**	1.808	1.206	1.513	0.159
**MRSA**	1.572	1.24	0.293	1.204
**MRSA**	2.032	1.174	2.671	1.431
**MRSA**	1.787	1.162	1.358	0.849
**MRSA**	2.085	1.2	2.98	1.162
**MRSA**	1.852	1.147	1.684	1.332

Table S8. Percentage of accurate classification of RAW 264.7 macrophages infected by different bacteria from Jackknifed analysis. The results show an overall 68% correct classification.

	Cell only	E. coli	MRSA	Correct (%)
**Cell only**	8	2	0	80
E. coli	3	4	2	44
**MRSA**	1	1	7	78
**Total**	12	7	9	68

References

[i]. Jiang, M.; Chattopadhyay, A.N.; Li, C.H.; Geng, Y.; Luther, D.C.; Huang, R.; Rotello, V.M. Direct Discrimination of Cell Surface Glycosylation Signatures Using a Single PH-Responsive Boronic Acid-Functionalized Polymer. *Chem. Sci.* **2022**, *29*, doi:10.1039/d2sc02116a.

[ii]. Landis, R.F.; Li, C.H.; Gupta, A.; Lee, Y.W.; Yazdani, M.; Ngernyuang, N.; Altinbasak, I.; Mansoor, S.; Khichi, M.A.S.; Sanyal, A.; et al. Biodegradable Nanocomposite Antimicrobials for the Eradication of Multidrug-Resistant Bacterial Biofilms without Accumulated Resistance. *J. Am. Chem. Soc.* **2018**, *140*, 6176–6182, doi:10.1021/jacs.8b03575.
